# Quality of life and factors associated among public university employees retired due to disabilities

**DOI:** 10.1590/1518-8345.6057.3816

**Published:** 2023-01-30

**Authors:** Aline Aparecida Oliveira Moreira, Júlia Trevisan Martins, Maria Lucia do Carmo Cruz Robazzi, Maria José Quina Galdino, Renata Perfeito Ribeiro, Maynara Fernanda Carvalho Barreto

**Affiliations:** 1 Universidade Estadual de Londrina, Departamento de Enfermagem, Londrina, PR, Brazil.; 2 Universidade de São Paulo, Escola de Enfermagem de Ribeirão Preto, PAHO/WHO Collaborating Centre for Nursing Research Development, Ribeirão Preto, SP, Brazil.; 3 Universidade Estadual do Norte do Paraná, Departamento de Enfermagem, Bandeirantes, PR, Brazil.

**Keywords:** Retirement, Insurance, Disability, Quality of Life, Occupational Health, Universities, Government Employees, Aposentadoria, Seguro por Invalidez, Qualidade de Vida, Saúde do Trabalhador, Universidades, Empregados do Governo, Jubilación, Seguro por Invalidez, Calidad de Vida, Salud Laboral, Universidades, Empleados del Estado

## Abstract

**Objective:**

to analyze quality of life and factors associated among public university employees retired due to disabilities.

**Method:**

a cross-sectional study conducted with a sample of public university employees retired due to disabilities. A characterization questionnaire and the World Health Organization Quality of Life - Disabilities instrument were applied via telephone or online contacts from November 2019 to September 2020. The associated factors were verified through multiple linear regression*.*

**Results:**

of the 80 retirees due to disability, 15% were professors and 85% had a technical-administrative career. As for the factors associated with Quality of Life, continuous medication use (β^adj^: -0.25; p=0.02) and problems in the nervous system (β^adj^: -0.21; p<0.05) were associated with the Overall domain; continuous medication use (β^adj^: -0.23; p=0.04), to the Physical domain; smoking (β^adj^: -0.21; p<0.05) and mental and behavioral disorders (β^adj^: -0.21; p<0.01), to the Psychological domain; smoking (β^adj^: -0.46; p<0.01) and respiratory (β^adj^: -0.21; p=0.03) and circulatory (β^adj^: -0.21; p=0.03) problems, to the Social domain; smoking (β^adj^: -0.33; p<0.01) and problems in the nervous system (β^adj^: -0.22; p=0.04), to the Environmental domain; mental and behavioral disorders, to the Disabilities module (β^adj^: -0.29; p<0.01) and to the Discrimination domain (β^adj^: -0.21; p<0.05); and smoking (β^adj^: -0.32; p<0.01) and problems in the nervous system (β^adj^: -0.20; p<0.05), to the Inclusion domain. The Autonomy domain did not present any association.

**Conclusion:**

the retirees under study presented impaired Quality of Life.

## Resumo

**Objetivo:**

analisar a qualidade de vida e os fatores associados entre servidores de universidades públicas aposentados por invalidez.

**Método:**

estudo transversal, com amostra de servidores aposentados por invalidez de universidades públicas. Um questionário de caracterização e o *World Health Organization Quality of Life - Disabilities* foram aplicados por contato telefônico ou *online* no período de novembro de 2019 a setembro de 2020. Verificaram-se os fatores associados por regressão linear múltipla*.*

**Resultados:**

dos 80 aposentados por invalidez, 15% eram docentes e 85% da carreira técnica-administrativa. Quanto aos fatores associados à qualidade de vida, o uso de medicação contínua (β^aj^: -0,25; p=0,02) e os problemas do sistema nervoso (β^aj^: -0,21; p<0,05) associaram-se ao domínio *Overall*; o uso de medicação contínua (β^aj^: -0,23; p=0,04) ao domínio físico; o tabagismo (β^aj^: -0,21; p<0,05) e os transtornos mentais e comportamentais (β^aj^: -0,21; p<0,01) ao domínio psicológico; o tabagismo (β^aj^: -0,46; p<0,01), os problemas respiratórios (β^aj^: -0,21; p=0,03) e circulatórios (β^aj^: -0,21; p=0,03) ao domínio social; o tabagismo (β^aj^: -0,33; p<0,01) e os problemas do sistema nervoso (β^aj^: -0,22; p=0,04) ao domínio ambiental; os transtornos mentais e comportamentais ao módulo incapacidades (β^aj^: -0,29; p<0,01) e ao domínio discriminação (β^aj^: -0,21; p<0,05); o tabagismo (β^aj^: -0,32; p<0,01) e os problemas do sistema nervoso (β^aj^: -0,20; p<0,05) ao domínio inclusão. O domínio autonomia não apresentou associação.

**Conclusão:**

os aposentados estudados apresentaram uma qualidade de vida prejudicada.

## Resumen

**Objetivo:**

analizar la calidad de vida y los factores asociados de empleados de universidades públicas jubilados por invalidez.

**Método:**

estudio transversal, con una muestra de trabajadores jubilados por invalidez de universidades públicas. Se aplicó un cuestionario de caracterización y el *World Health Organization Quality of Life - Disabilities* mediante contacto telefónico u *online* desde noviembre de 2019 hasta septiembre de 2020. Los factores asociados se verificaron mediante regresión lineal múltiple.

**Resultados:**

de los 80 jubilados por invalidez, el 15% era docente y el 85% era técnico-administrativo. En cuanto a los factores asociados a la calidad de vida, el uso continuo de medicamentos (β^aj^: -0,25; p=0,02) y los problemas del sistema nervioso (β^aj^: -0,21; p<0,05) se asociaron al dominio *overall*; el uso continuo de medicamentos (β^aj^: -0,23; p=0,04) el dominio físico; el tabaquismo (β^aj^: -0,21; p<0,05) y los trastornos mentales y conductuales (β^aj^: -0,21; p<0,01) al dominio psicológico; el tabaquismo (β^aj^: -0,46; p<0,01), los problemas respiratorios (β^aj^: -0,21; p=0,03) y circulatorios (β^aj^:-0,21;p=0,03) al dominio social; el tabaquismo (β^aj^: -0,33; p<0,01) y los problemas del sistema nervioso (β^aj^: -0,22; p=0,04) al dominio ambiental; los trastornos mentales y conductuales al módulo discapacidad (β^aj^: -0,29; p<0,01) y al dominio discriminación (β^aj^: -0,21; p<0,05); el tabaquismo (β^aj^: -0,32; p<0,01) y los problemas del sistema nervioso (β^aj^: -0,20; p<0,05) al dominio inclusión. El dominio autonomía no mostró asociación.

**Conclusión:**

la calidad de vida de los jubilados por invalidez que participaron del estudio estaba deteriorada.

**Highlights**

(1) Mental disorders negatively interfere with quality of life.

(2) Retirees due to disabilities present impaired quality of life.

(3) Advanced age is associated with lower quality of life scores.

## Introduction

Advances in science and technology drive transformation in societies, institutions and world economies, changing the ways of living, working and interacting^1^, which can exert positive impacts on people’s Quality of Life (QoL) or impair it. In addition, they lead to changes in the demographic sphere, with a decrease in the birth rate and an increase in life expectancy, as well as in the epidemiological context, with an increase in morbidity and mortality due to chronic non-communicable diseases (CNCDs), representing a challenge for the countries in the elaboration of public policies that improve and prolong workers’ lives, thus maintaining the financial balance of the different social security systems^2^ ^-^ ^3^.

In addition to that, the morbidity and mortality due to CNCDs causes high prevalence of deaths, limitations and disabilities, as well as it reduces individuals’ QoL around the world, especially in emerging countries and even responsible for premature deaths^4^.

Added to the evolution of Information and Communication Technologies (ICTs), the current organization of capitalist society has transformed and innovated work organization and labor relations and increased productivity, representing a true paradigm shift. While this can be beneficial in several aspects, it also affected work-life balance and triggered increasingly stressful working modalities for physical, mental and social health^5^. Such factors can lead to illness, with a consequent temporary or even definite distancing of the professionals from their job. Distancing due to diseases can culminate in disability retirement due to the high impairment level, exerting impacts on the professional area and on public health, in addition to the harms to the workers^6^.

Retirement motivated by disability is the one granted to insured workers who become definitively unable to work due to illness or accident, after proof by medical expertise. The benefits are proportional to the workers’ contributions, except in case of work-related accidents, professional diseases or serious, communicable or incurable diseases^7^.

It should be noted that retirement can give rise to multiple transformations in the life of a human being, that is, it is a complex paradox, as it can mean freedom for workers, being a coveted moment in which they will have time to take care of personal projects, of their family and of themselves. However, it can have a negative connotation, if presented as a time to retire, withdraw into one’s quarters, of finitude, abandonment, inactivity, being left aside by society^8^.

When this exit is due to a disability situation, there will be consequences both in the workforce and in people’s everyday life, in addition to the impacts on QoL. A study showed that the better the perception of QoL during the professional career, the greater the desire not to dissociate from work^9^. Retirement is a singular transition period intrinsic to the aging process, and its association with higher satisfaction with life can exert positive effects on overall health, functioning and QoL. However, people who retire due to disabilities undergo a particular experience and differ from those that chose to retire based on service time or age. Such being the case, it can be inferred that disability retirement can impose harms to these people’s QoL.

The concept of QoL adopted in this study is related to subjective aspects related to social, cultural and environmental issues, and can be defined from how individuals perceive their life in the context in which they are inserted in terms of culture, values, goals, expectations, rules and concerns^10^.

Given the above, this study is relevant because may provide reflections on the QoL of people retired due to disability, providing subsidies for the implementation of measures that seek to prevent and promote the workers’ health and, consequently, avoid early retirement. It is worth emphasizing that the studies evaluating the relationship between retirement and life satisfaction were carried out in high-income countries such as the United States and Europe, and that few were carried out in low- or middle-income countries such as Brazil, in which the living conditions and social protection schemes are more unfavorable^11^. The objective of this research study was to analyze Quality of Life and factors associated among public university employees retired due to disabilities.

## Method

### Type of study

This is a cross-sectional study with a quantitative approach. As such, description of this section followed the recommendations set forth in Strengthening the Reporting of Observational Studies in Epidemiology (STROBE).

### Study population and locus

The population consisted of retired employees due to disabilities from 2007 to 2017, from seven public universities of the state of Paraná (PR), Brazil. The period selected refers to the inauguration of one of the seven universities under study (2007), as well as to the date of the last disability retirement granted in the research sites (2017). 

### Selection criteria

The eligibility criteria were as follows: being retired due to disability for at least one year, having worked under a statutory regime in one of the universities, and being able to answer the questionnaires either in writing or verbally. 

### Sample

A total of 150 possible participants were identified. The sample consisted of all the employees retired due to disability that were located and who agreed to participate in the research, totaling 80 respondents, with representatives from all the eligible universities, with 01 to 56 participants *per* university. 

### Instruments used and study variables

The data were collected through a self-report instrument, whose first part contained questions to obtain sociodemographic data (gender, age, marital status, schooling and family income), occupational information (function held before retirement, year of retirement, length of service at the university and weekly workload), clinical data (presence of comorbidities, continuous medication use and causes of permanent disability), and life habits (alcohol consumption, smoking and physical activity). These characterization questions were prepared by the first author and submitted to a pilot test with seven employees retired due to disability from two public universities in other Brazilian states, which proved to be adequate to achieve the objective proposed.

The second part evaluated QoL, in which the World Health Organization Quality of Life instrument was selected, a tool used worldwide at no cost and developed by the World Health Organization (WHO), with a specific assessment module for people with disabilities: World Health Organization Quality of Life - Disabilities (WHOQOL-DIS).

The WHO recommends applying the World Health Organization Quality of Life Assessment Instrument - Bref (WHOQOL-Bref), which evaluates QoL through 24 facets divided into four domains (Physical, Psychological, Social Relationships and Environment) and two questions for the general assessment of QoL (Overall)^12^. Subsequently, the *Disabilities* module (DIS Module), consisting of 13 facets, one for the general evaluation of the impact of QoL deficit, and the others divided into three domains: Discrimination, Autonomy and Inclusion (Figure 1)^13^. Therefore, WHOQOL-DIS consists in applying WHOQOL-Bref in addition to the DIS Module^14^.

**Figure 1:** Distribution of the facets from the WHOQOL-Bref domains and the Disabilities Module

Domains	Facets
*World Health Organization Quality of Life Assessment Instrument - Bref (WHOQOL-Bref)*	
Overall	Overall quality of life /
	Overall health
I. Physical	Pain and discomfort
	Dependence on medications or treatments
	Energy and fatigue
	Mobility
	Sleep and rest
	Activities of daily living
	Work capacity
II. Psychological	Positive feelings
	Spirituality, religion and personal beliefs
	Thinking, learning, memory and concentration
	Bodily image and appearance
	Self-esteem
	Negative feelings
III. Social Relationships	Personal relationships
	Sexual activity
	Social support
IV. Environment	Physical safety and security
	Physical environment (pollution/noise/traffic/climate)
	Financial resources
	Opportunities for acquiring new information and skills
	Participation in and opportunities for recreation/leisure activities.
	Home environment
	Health and social care: accessibility and quality
	Transportation
*Disabilities Module (DIS Module)*	
Overall-DIS	1. Impact of the disability
I - Discrimination	Discrimination
	Protection
	Future perspectives
II. Autonomy	Control of your life
	Decision-making power
	Autonomy
III. Inclusion	Communication ability
	Social acceptance
	Respect
	Social interaction
	Social inclusion
	Personal capacity

Source: World Health Organization (1996)^12^; The WHOQOL-DIS Group (2011)^13^

WHOQOL-Bref and the DIS Module were translated and validated to Brazilian Portuguese in 2000 and 2014, respectively^14^ ^-^ ^15^. The WHOQOL-DIS has answers provided on five-point Likert scales and does not have a cutoff score; however, the scores obtained in the domains must be transformed into a scale from 0 to 100, where the closer to zero, the worse the QoL, and the closer to 100, the better the QoL^12^ ^-^ ^13^.

### Data collection and period

Data collection w6as carried out by the first author, where the participants were identified by means of the data provided by the universities and invited to participate via phone calls, electronic messages and dissemination on the website of one of the universities. The questionnaires were answered online *via Google Forms* or through telephone contacts, according to the participants’ preferences, from November 2019 to September 2020. Regarding the 46.6% (n=70) population loss, 20% (n=30) died and 26.6% (n=40) refused to participate. 

### Data analysis

The Statistical Package for the Social Sciences (SPSS) program, version 20.0, was used for the statistical analyses. The variables were described using central tendency and variability measures or through absolute and relative frequencies.

The dependent variables of the study presented normal distribution, as indicated by the Kolmogorov-Smirnov test (p>0.05). The association of the WHOQOL-DIS dimensions and facets with the independent variables was verified by resorting to Pearson’s correlation coefficient. In order to define the set of variables that best explained the outcome, a multiple regression analysis was performed, through the “forward bootstrap” method. Sample size was considered adequate for this analysis, as the literature recommends from 10 to 15 cases for each variable included in the model, with a minimum of 50 cases^16^. All the assumptions of this analysis method were met and, for the collinearity diagnoses, Variance Inflation Factor (VIF) values from 1.008 to 2.110 were obtained, which was considered adequate. The statistically significant variables were maintained, as well as those that adjusted β1 in at least 10%. All the models were adjusted by gender and age, for being considered potential confounders of the relationship. A 5% significance level and 95% confidence intervals were considered for all the tests.

### Ethical aspects

The current ethical precepts were followed in this research, and approval was obtained from the Research Ethics Committee after authorization from the state universities, with Certificate of Presentation of Ethical Appreciation (*Certificado de Apresentação de Apreciação Ética*, CAAE) number 03990518.5.0000.5231. The respondents who participated through electronic media had access to the instruments after agreeing with and signing the Free and Informed Consent Form (FICF) and, via the telephone contacts, such consent was first read and then recorded.

## Results

Of the 150 employees retired due to disability eligible for the study, 80 agreed to participate, 15.0% were higher education professors and 85.0% were administrative technicians, belonging to the higher (5.0%), medium (43.8%) %) and operational (36.3%) levels. Most of them were female (65%), had no marital relationships (77.5%), at least one current health problem (93.8%) and were on continuous medication use (92.5%). Age and retirement time presented mean values of 59.83 (±8.98) and 8.31 (±3.76) years, respectively. According to the mean dollar exchange rate (US$ 1 = R$ 5.09) in 2020^17^, monthly income varied between US$ 235.76 (R$ 1,200.00) and US$ 6,876.23 (R$ 35,000.00), with a mean of US$ 1,073.92 (R$ 5,466.25). Regarding life habits, 10% presented excessive consumption of alcoholic beverages, 65% were sedentary and 23.8% smoked.

The WHOQOL-DIS descriptive measures are shown in Table 1, with the highest and lowest mean values in the Autonomy and Physical domains, respectively.

**Table 1:** WHOQOL-DIS^*^ descriptive measures of the state public university employees retired due to disabilities (n=80). Paraná, Brazil, 2019-2020

WHOQOL-DIS^*^	Mean	Standard Deviation	Median	Interquartile Range
Overall	50.16	28.02	50.00	25.00-75.00
Physical	46.74	21.40	46.43	28.57-64.29
Psychological	54.64	23.31	54.17	33.33-73.96
Social Relationships	55.83	28.11	54.17	33.33-75.00
Environment	62.89	15.69	62.50	53.13-71.88
Overall*-*Disabilities	63.43	20.57	67.71	47.92-81.25
Discrimination	65.78	23.56	66.67	50.00-83.33
Autonomy	75.99	25.30	83.33	66.67-100.00
Inclusion	55.94	23.73	58.33	37.50-70.83

* World Health Organization Quality of Life - Disabilities

The WHOQOL-DIS domains presented correlations with the sociodemographic characterization, clinical and occupational variables, as described in Table 2.

**Table 2:** p-values^*^ corresponding to the Pearson’s correlations of WHOQOL-DIS^†^ with the characterization variables of the state public university employees retired due to disabilities (n=80). Paraná, Brazil, 2019-2020

Variables	Overall	Physical	Psychological	Social Relationships	Environment	**Overall-*Disabilities***	Discrimination	Autonomy	Inclusion
Age	0.22	0.32	<0.01	0.06	0.01	0.08	0.27	0.70	0.02
Gender	<0.01	0.03	<0.01	0.16	0.30	0.03	<0.01	0.23	0.10
Marital status	0.22	0.46	0.11	0.02	0.20	0.30	0.48	0.55	0.08
Having dependents	0.88	0.45	0.11	0.33	0.50	0.47	0.28	0.93	0.06
Monthly income	0.40	0.52	0.73	0.88	0.07	0.72	0.24	0.85	0.89
Excessive alcohol consumption	0.19	0.26	0.59	0.35	1.00	0.96	0.61	0.81	0.98
Physical activity	0.19	0.60	0.21	0.23	0.17	0.58	0.57	0.61	0.68
Smoking	0.15	0.26	<0.01	<0.01	<0.01	0.02	1.00	0.20	<0.01
Type of professional career	0.26	0.71	0.25	0.55	0.35	0.53	0.41	0.86	0.56
Genitourinary problems	0.17	0.46	0.16	0.28	0.33	0.53	0.66	0.80	0.46
Musculoskeletal problems	0.39	0.13	0.68	0.43	0.50	0.48	0.74	0.46	0.34
Respiratory problems	0.34	0.18	0.15	<0.05	0.14	0.32	0.65	0.60	0.22
Circulatory problems	0.96	0.68	<0.01	<0.01	0.12	0.08	0.32	0.83	0.01
Problems in the eyes	0.15	0.97	0.15	0.25	0.90	0.17	0.93	0.08	0.15
Problems in the nervous system	0.03	0.46	0.01	0.13	<0.01	0.03	0.10	0.23	0.02
Mental and behavioral disorders	0.16	0.36	<0.01	<0.01	0.04	<0.01	0.02	0.20	<0.01
Endocrine or nutritional problems	0.35	0.70	<0.05	0.28	0.87	0.42	0.77	0.84	0.25
Infectious or parasitic diseases	0.20	0.28	0.59	0.77	0.38	0.62	0.65	0.45	0.82
Neoplasms	0.17	0.62	0.85	1.00	0.45	0.77	0.99	0.98	0.61
Continuous medication use	0.06	0.02	0.12	0.27	0.60	0.15	0.04	0.55	0.26

* Pearson’s correlation coefficient; ^†^World Health Organization Quality of Life - Disabilities

The multiple models of the WHOQOL-DIS domains are presented in Table 3.

**Table 3:** Multiple unadjusted and adjusted linear regression models of the WHOQOL-DIS^*^ dimensions and facets among the state public university employees retired due to disabilities (n=80). Paraná, Brazil, 2019-2020

Multiple models^†^	Beta	p-value	95% Confidence Interval		Beta	p-value	95% Confidence Interval	
*Overall (R* ^*2*^ *=0.201)*								
Problems in the nervous system	-0.26	0.02	-4.17	-46.52	-0.25	0.02	-4.39	-45.44
Continuous medication use	-0.23	0.03	-47.47	-2.03	-0.21	<0.05	-44.12	-0.31
Gender					0.11	0.30	-5.52	17.67
Age					-0.27	0.01	-3.78	-28.01
*Physical (R* ^*2*^ *=0.115)*								
Continuous medication use	-0.25	0.03	-37.87	-2.64	-0.23	0.04	-36.00	-1.15
Gender					-0.05	0.65	-11.30	7.05
Age					-0.23	0.04	-0.52	-19.83
*Psychological (R* ^*2*^ *=0.302)*								
Mental and behavioral disorders	-0.28	0.01	-24.28	-3.03	-0.21	<0.05	-20.77	-0.08
Smoking	-0.27	0.02	-26.67	-2.85	-0.31	<0.01	-28.57	-5.62
Gender					0.15	0.12	-1.84	16.16
Age					-0.25	0.01	-2.73	-21.74
*Social Relationships (R* ^*2*^ *=0.378)*								
Smoking	-0.46	<0.01	-42.64	-17.63	-0.465	<0.01	-43.07	-17.93
Respiratory problems	-0.23	0.02	-74.24	-7.19	-0.214	0.03	-71.99	-4.55
Circulatory problems	-0.22	0.03	-1.53	-23.12	-0.21	0.03	-0.99	-22.74
Gender					0.03	0.78	-8.90	11.80
Age					0.13	0.16	-3.11	18.52
*Environment (R* ^*2*^ *=0.234)*								
Smoking	-0.31	<0.01	-19.14	-3.87	-0.33	<0.01	-19.77	-4.73
Problems in the nervous system	-0.24	0.03	-1.71	-24.71	-0.22	0.04	-0.60	-23.40
Gender					0.19	0.07	-0.54	12.23
Age					0.12	0.25	-2.75	10.48
*Overall-Disabilities (R* ^*2*^ *=0.161)*								
Mental and behavioral disorders	-0.33	<0.01	-23.21	-4.97	-0.29	<0.01	-21.56	-3.39
Gender					0.12	0.26	-3.69	13.51
Age					-0.19	0.08	-0.96	-17.24
*Discrimination (R* ^*2*^ *=0.169)*								
Mental and behavioral disorders	-0.27	0.02	-23.73	-2.40	-0.21	<0.05	-20.87	-0.14
Gender					0.10	0.36	-5.31	14.31
Age					-0.29	<0.01	-4.07	-24.82
*Inclusion (R* ^*2*^ *=0.246)*								
Smoking	-0.29	0.01	-28.07	-3.60	-0.32	<0.01	-29.80	-5.51
Mental and behavioral disorders	-0.25	0.03	-23.13	-1.30	-0.20	<0.05	-20.88	1.03
Gender					0.16	0.13	-2.18	16.87
Age					0.15	0.16	-2.82	17.29

* World Health Organization Quality of Life - Disabilities; ^†^There was no model with significant variables for the Autonomy domain from WHOQOL-DIS

It was verified that overall QoL was reduced for the retirees due to problems in the nervous system and on continuous medication use, especially in the older ones. Physical QoL was significantly reduced for the retirees due to Mental and Behavioral Disorders (MBDs), mainly among those of more advanced age. Psychological QoL was significantly reduced for the smokers and retirees due to MBDs, also associated with increased age. Social QoL was significantly reduced for smokers and retirees due to respiratory and circulatory problems, regardless of age and gender. Environmental QoL was also reduced for smokers and retirees due to problems in the nervous system, regardless of age and gender.

Overall QoL related to disability was reduced for the retirees due to MBDs, especially in the older ones. In the Discrimination domain, those who retired due to MBDs and of more advanced age presented lower QoL levels. Similarly, retirees due to MBDs and smokers obtained lower scores in the Inclusion domain.

## Discussion

The retirees due to disabilities under study presented lower QoL scores. The main factors that were associated with the lower QoL scores were MBDs and problems in the nervous system, as reasons for retirement, as well as the smoking habit.

The prevalence of disability retirement is associated with the high demands in terms of physical work and with the deficient psychosocial conditions at work^18^ ^-^ ^19^. There is diverse evidence about high demands and low control at work as predictors for disability retirement^20^.

It is believed that the public service structure and organization are obstacles for workers to control the work performed, contributing to early retirements. These data are similar to those from another study conducted with retirees due to disabilities, which identified that they were still individuals of productive age^21^. This fact exerts impacts on the Brazilian social security system, which has a history of deficits, as well as on financial deficits for workers, who often did not reach the necessary contribution time to realize full retirement^22^.

Age below the established for retirement among those distanced due to disabilities was identified in different realities, with various groups of workers around the world^21^ ^,^ ^23^. This fact only ratifies the importance of this theme and of implementing measures to prevent early exit from the labor market.

The high frequency of health problems and medication use is due to the very disability retirement resulting from inability to work caused by the evolution of the pre-existing diseases. There is evidence that the time of sick leave as active worker is a predictor of disability retirement, regardless of the activity performed^24^, and it is necessary and urgent to promote interventions with the workers while they are active to prevent them from advancing towards permanent disability.

Life habits are also determinants for health and QoL. Overweight, smoking, alcohol abuse, sleep disorders and limited physical activity are associated with more disease burden, absenteeism and disability retirement^25^ ^-^ ^26^.

When comparing the WHOQOL-DIS scores obtained by retirees due to disability in this study to the Brazilian normative data from the general population of southern Brazil^27^, it was verified that the retirees under study presented lower scores in all the domains, indicating that disability retirement can lead to impairments in people’s QoL.

Despite the limitations that culminated in disability, the retirees surveyed obtained higher scores in the Autonomy domain in the assessment of disabilities, that is, the respondents consider themselves in control of their lives and respected by those around them. A systematic review on the factors associated with autonomy in older adults identified its multifactorial and biopsychosocial character, with better physical and mental QoL, age group between 60 and 69 years old, satisfaction with life and family relationships among its positive associations^28^.

The aging process promotes a progressive decrease of the biological functions and favors emergence of CNCDs, as well as of the complications resulting from lack of adequate control of health problems throughout life. The treatment defined often implies more than two medications. Polymedication impairs the individual’s QoL and functional capacity since, in addition to the adverse reactions caused by drug interactions, it increases the risk of falls and, consequently, family dependence^29^ ^-^ ^30^. In this study, medication use was related to QoL deterioration associated with increased age, in line with the literature.

With regard to smoking, it is known that it is costly for public health, as it is associated with preventable deaths and constitutes an important risk factor for respiratory and cardiovascular diseases and cancer; in addition, smoking history and the degree of nicotine dependence are related to QoL deterioration^31^. Such assertion is in line with the results of the current study, as smoking contributed to lower scores in psychological, social and environmental QoL, as well as regarding inclusion of retirees due to disability, being a modifiable life habit and subjected to interventions to overcome the addiction and, thus, collaborate to improving QoL in retirees due to disability under study.

A study carried out in China showed that, for smokers, the mean chances of enjoying better QoL were 11.65% lower than when they did not smoke, highlighting the need for anti-smoking campaigns to clearly indicate the negative effect of tobacco use on people’s QoL^32^.

In the current study, the QoL domains affected by MBDs were not related to gender, although they were associated with increased age. A Spanish study that evaluated the QoL of people with severe mental illness identified that women’s overall mean QoL in the physical component was significantly lower than that of men, and that the factors associated with QoL also differed by gender^33^.

It should be noted that not all people affected by MBDs need to retire, and the universities under study have a work readaptation process, with functions compatible with their health condition assigned without loss of profits. Such being the case, only those unable to work retire due to disabilities. In addition, whether retired or readapted, people with MBDs need to be reintegrated into the citizenship process and its basic requirements, that is, accessibility to social rights such as health, education, social assistance, social security, housing, work and income, food security, mobility and public transportation and access to social, cultural, sports and tourism, leisure and digital inclusion opportunities, as provided for in Resolution No. 8 of the National Human Rights Council (*Conselho Nacional dos Direitos Humanos*, CNDH) which provides for MBDs and drug and alcohol users, published on August 14^th^, 2019, a rule intended to guide mental health policies and/or those related to problematic use of alcohol and other drugs throughout the national territory with a special focus on state agents and institutions^34^. 

The impact of health problems in the respiratory, circulatory and nervous systems on workers’ QoL reinforces the need for measures to promote health and prevent complications related to CNCDs, as they are associated with lower QoL^27^ ^,^ ^35^.

The influence of increased age on the lower QoL scores among the studied population stands out, indicating the need for measures prior to aging, as the reduction in functional capacity is not only linked to chronological age but is significantly influenced by the life context of the individual, finding throughout this path modifiable factors that can be worked on in order to contribute to healthy aging and well-being, in advanced age^36^.

It is worth noting the importance of the workers’ health knowledge area, in the case of diversified and complex studies that intervene in the factors that protect workers’ health and in those that can influence their illness, from a preventive perspective and targeted at improving the working conditions. A range of factors interfere with people’s health, including aging and its inherent comorbidities. Therefore, it is necessary that managers pay attention to the inherent needs of this population segment and devise strategies targeted at improving their QoL, especially when they are carrying out their work activities aiming at health promotion and disease prevention^37^.

This research presents limitations related to the method, precluding generalization of the results. Other limits concern the scarce literature on the theme of QoL with retirees due to health reasons and the COVID-19 pandemic, making it difficult to collect face-to-face data, which may have favored the refusals to participate. However, the study advances and brings about significant academic and social contributions, as it shows aspects of disability retirement that led to harms in the QoL of employees of state public universities. It is also revealed that there are modifiable factors that can be worked on prior to inability to work. It is suggested to conduct more studies on the topic to better understand the phenomenon in the national territory.

## Conclusion

The study participants showed impairments in their QoL, and medication use, smoking, retirements due to problems in the nervous, circulatory and respiratory systems and, mainly, MBDs, were negatively associated in several QoL domains, regardless of gender although they were associated with increasing age in some domains.

Thus, it is reinforced that it is necessary to plan and implement public policies to improve workers’ QoL, avoiding disability retirement, also emphasizing that local managers, together with workers, must seek actions to prevent diseases and health problems and, in turn, increase the chances of health promotion and well-being, preventing early retirement.
